# IgG4-related disease involving vital organs diagnosed with lip biopsy: A case report and literature review

**DOI:** 10.1097/MD.0000000000003970

**Published:** 2016-06-17

**Authors:** Mitsuhiro Akiyama, Yuko Kaneko, Yutaro Hayashi, Tsutomu Takeuchi

**Affiliations:** Division of Rheumatology, Department of Internal Medicine, Keio University School of Medicine, Tokyo, Japan.

**Keywords:** diagnosis, IgG4-related disease, lip biopsy, treatment

## Abstract

Immunoglobulin G4-related disease (IgG4-RD) is a recently recognized new disease entity characterized by elevated serum IgG4 and infiltration of IgG4^+^ plasma cells in affected tissues. Histological examination is essential for definitive diagnosis, as other pathological conditions can also present with serum IgG4 elevation. However, IgG4-RD frequently involves vital or internal organs that are difficult to perform biopsies. We herein report a unique case of IgG4-RD involving vital organs that could be successfully diagnosed by alternative lip biopsy, an accessible, little invasive procedure, despite no apparent manifestation demonstrating the involvement in labial salivary gland.

A 60-year-old man with swelling of both submandibular glands and elevated serum creatinine level visited our hospital. His labial salivary glands appeared normal. His blood test showed high serum IgG4, and positron-emission computed tomography revealed abnormal uptake in submandibular glands, periaorta, and left kidney with hydronephrosis. We suspected him of IgG4-RD; however, the involved organs were difficult to approach for histological examination. Alternatively, we performed lip biopsy and proved massive infiltration of IgG4^+^ plasma cells leading to the diagnosis with IgG4-RD. Treatment with prednisolone resulted in the remarkable improvement of organ involvements and the normalization of serum IgG4 level after 3 months. Prednisolone was gradually tapered without the relapse of disease.

The early recognition and diagnosis of IgG4-RD is clinically important because delay in the treatment initiation leads to fibrosis with irreversible organ damage. Our case highlights the possibility that lip biopsy is a promising option for histological examination in patients with IgG4-RD in whom affected organs are difficult to access, leading to early diagnosis with appropriate treatment.

## 1 Introduction

Immunoglobulin G4-related disease (IgG4-RD) is a new disease entity characterized by elevated serum IgG4 and infiltration of IgG4^+^ plasma cells into various organs.^[1–4]^ Because serum IgG4 could be elevated in other pathologic conditions, biopsies of local affected lesions are recommended for definitive diagnosis of IgG4-RD.^[5–9]^ Umehara et al have proposed the 2011 comprehensive diagnostic criteria, and recommended that the diagnosis should be pathologically confirmed.^[10]^ More recently, international consensus guidance statement on the management and treatment of IgG4-RD has also strongly recommended the diagnostic confirmation by biopsy for the exclusion of malignancies and other IgG4-RD mimics.^[11]^ However, vital organ involvement sometimes causes difficulty in obtaining specimen necessary for the pathological diagnosis. Delay in the diagnosis and the treatment initiation leads to fibrosis with irreversible organ damage in this disease. In this article, we report a unique case of IgG4-RD involving vital, inaccessible organs that could be successfully diagnosed with alternative lip biopsy, leading to the early diagnosis with appropriate treatment.

## 2 Case presentation

A 60-year-old man with past history of allergic rhinitis noticed the swelling of bilateral submandibular glands (SMGs) in June 2014. Serum creatinine elevation (1.37 mg/dL) was pointed out at routine health checkup around the time and was admitted to our hospital for work-up.

Physical examination revealed normal blood pressure 127/83 mm Hg and body temperature 36.6°C. The findings of ocular, lung, cardiovascular, abdominal, neurological, and skin examination were normal. Although his bilateral SMGs were swelling with no tenderness, his labial salivary gland (LSG) appeared normal.

Laboratory tests revealed elevated serum creatinine (1.78 mg/dL, normal range: 0.61–1.10 mg/dL), IgG (1856 mg/dL, normal range: 870–1700 mg/dL), IgG4 (314 mg/dL, normal range: 4.8–105 mg/dL), and slightly elevated C-reactive protein (0.74 mg/dL, normal range: <0.30 mg/dL). Other blood tests, including blood count, serum electrolyte levels, liver enzyme levels, and blood glucose were within normal ranges. Antinuclear antibody, anti-SS-A, and SS-B antibodies were all negative. Urinalysis showed neither proteinuria, occult blood, white blood cells nor casts.

**Figure 1:**
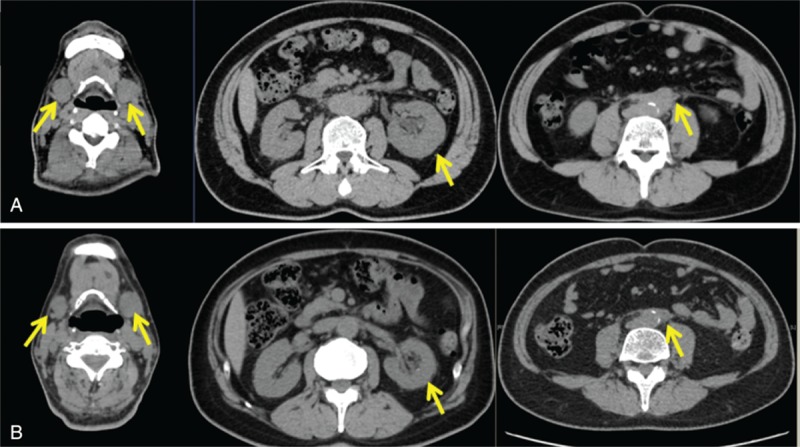
Computed tomography (CT) findings before and after glucocorticoids therapy: (A) CT findings before glucocorticoid therapy and (B) CT findings after glucocorticoid therapy. The swelling of submandibular glands (left), periaortitis (right), and left kidney (middle) was improved after glucocorticoids therapy (yellow arrows).

**Figure 2:**
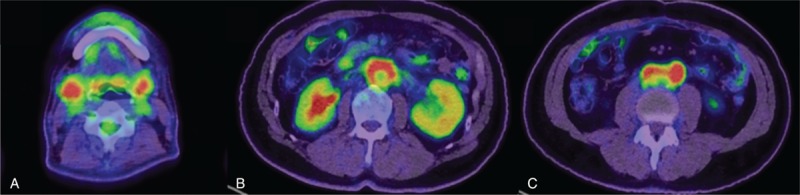
Positron-emission computed tomography findings. The abnormal accumulation was shown in submandibular glands (A), kidney (B), and periaorta (B, C).

Computed tomography (CT) revealed enlarged bilateral SMGs and the left kidney, the left hydronephrosis, and high-density area at the periaorta soft tissue (Fig. 1A). Positron-emission tomography-CT demonstrated abnormal accumulation of fluoro-2-deoxyglucose-positron at the identical area (bilateral SMGs, left kidney, and periaorta) (Fig. 2).

**Figure 3:**
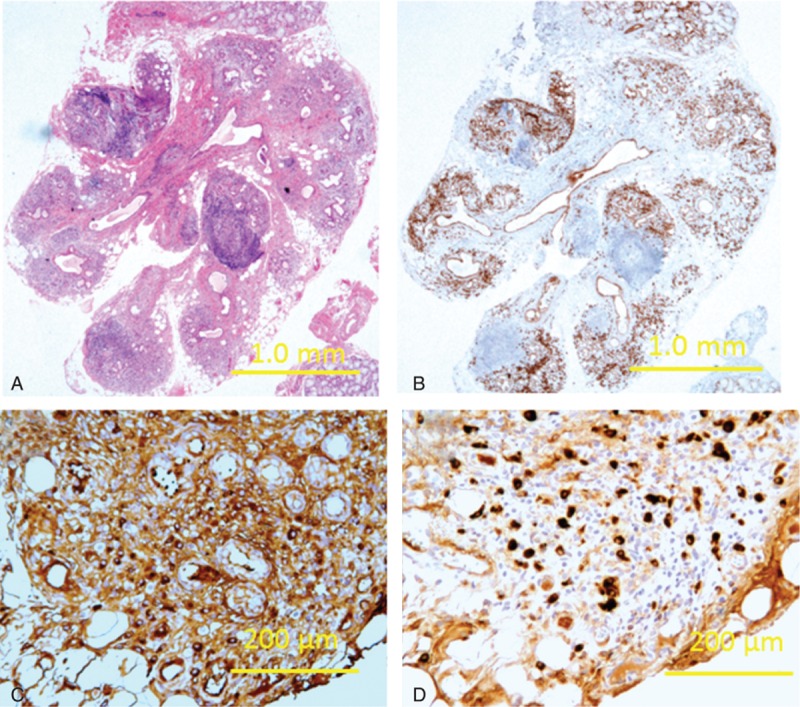
Histopathological findings of lip biopsy: (A) H&E staining reveals massive lymphocytic infiltration and germinal centre formation; (B) CD138 staining reveals massive plasma cells infiltration; (C) IgG immunostaining; and (D) IgG4 immunostaining. The ratio of IgG4^+^ plasma cells/IgG^+^ plasma cells was 47%.

IgG4-RD was suspected; however, biopsy of the periaorta had risk of the injury of abdominal aorta, and kidney biopsy was contraindicated owing to hydronephrosis. He did not agree on submandibulectomy for fear surgical risk. A fine needle biopsy of SMG would be inadequate for sampling in IgG4-RD patients.^[12]^ Thus, we performed less invasive lip biopsy in an attempt to obtain pathological specimen with patient agreement. Histological examination of his LSG revealed heavy infiltration of lymphocytes and hyperplastic germinal center formation (Fig. 3A), adding massive plasma cells infiltration revealed by CD138 staining (Fig. 3B). Forty-seven percent of those plasma cells with IgG immunoreactivity (Fig. 3C) were positively immunolabeled with antibody for IgG4 (Fig. 3D).

We diagnosed him as IgG4-RD based on the 2011 comprehensive diagnostic criteria,^[10]^ and treatment with 60 mg/d (0.8 mg/kg) of prednisolone was initiated. Three months later, although the reevaluation of LSG tissue after glucocorticoid treatment was not conducted, the swelling of SMGs and left kidney and periaortitis in CT remarkably improved (Fig. 1B). During follow-up of 14 months, serum levels of creatinine normalized to 1.10 mg/dL and IgG4 to 74 mg/dL. The dose of prednisolone was gradually tapered, and he has been treated with 5 mg daily without recurrence.

## 3 Discussion

The present case suggested that LSG tissues obtained by lip biopsy can be one of the options for the pathological diagnosis of IgG4-RD when performing biopsies of underlying organs is difficult, even though physical and image examinations did not suggest the LSG involvement.

The importance of pathological examination for diagnosis of IgG4-RD has been increasingly recognized because other diseases mimicking IgG4-RD sometimes present with elevated serum IgG4.^[5–7]^ However, tissues from deep organs such as pancreas, retroperitoneum, kidney, ocular cavity, and periaorta are difficult to perform biopsy. In addition, IgG4-RD mainly affects middle-aged to elderly men who often have comorbidities, which places them at risk for complications. As was the present case, when the difficulty, the contraindication, or the risk in access to affected organs is tangible, lip biopsy could be tried for the definitive diagnosis of IgG4-RD because lip biopsy is less invasive and easy to perform.

Although the positivity of lip biopsy in our case might have been accidental, some reports have revealed that it is worthwhile to pay attention on the usefulness of lip biopsy. We previously reported that the sensitivity of lip biopsy for diagnosis of IgG4-RD was 83.3%.^[13]^ Abe et al also reported the sensitivity of lip biopsy as 63.0%, and more recently, Moriyama et al reported the sensitivity of lip biopsy as 55.6% for IgG4-RD.^[14,15]^ Likewise, a case with only retroperitoneal fibrosis without Mikulicz's disease was successfully diagnosed by a lip biopsy.^[16]^ Another case report also indicated the usefulness of lip biopsy to support the diagnosis of IgG4-RD.^[17]^ In the previous study, 40.0% of IgG4-RD patients without lesions of salivary glands were positive for lip biopsy, and the mean number of affected organs and serum IgG4 levels in the positive cases were significantly higher than in the negative cases.^[15]^ Our present case also had multiple organ involvements, suggesting the association between the positivity of lip biopsy and the number of affected organs. Taken together, a certain amount of patients with IgG4-RD could be pathologically diagnosed by lip biopsy.

The international statement regarding the management and treatment of IgG4-RD has been recently published.^[11]^ The initiation of treatment was recommended for all patients with active IgG4-RD. Importantly, the organ involvement could be subclinical through the late stages of IgG4-RD, by which time chronic inflammation with fibrotic change might have led to the irreversible organ injury. Thus, it is recognized that the early intervention is important for the prevention of irreversible organ injury associated with progressive fibrosis in the salivary glands and pancreas lesions.^[18,19]^ In the present case, we successfully diagnosed him with IgG4-RD by lip biopsy, and treatment with glucocorticoid led to the improvement of his renal function, swelling of SMGs, and periaortitis. As patients with IgG4-RD usually respond well to treatment with glucocorticoid,^[11,20]^ early recognition and diagnosis is clinically important to prevent the irreversible organ injury.

## 4 Conclusions

In summary, we report a case of IgG4-RD affecting SMGs, periaorta, and kidney that could be pathologically diagnosed by lip biopsy. With lip biopsy being minimally invasive, convenient, and useful for pathological confirmation for IgG4-RD, we emphasize this procedure as one of the options when affected organs are difficult to approach. Early recognition and diagnosis of this disease is clinically important because most patients respond well to glucocorticoid, which prevents irreversible organ damage.
